# Biotransformation of Flavonoids by Newly Isolated and Characterized *Lactobacillus pentosus* NGI01 Strain from Kimchi

**DOI:** 10.3390/microorganisms9051075

**Published:** 2021-05-17

**Authors:** Chan-Mi Park, Gyoung-Min Kim, Gun-Su Cha

**Affiliations:** 1 School of Biological Sciences and Technology, Chonnam National University, 77 Yongbongro, Gwangju 61186, Korea; cmpark0710@gmail.com; 2 Department of Research and Development, Namhae Garlic Research Institute, 2465-8 Namhaedaero, Namhae 52430, Korea; policecop@hanmail.net

**Keywords:** biotransformation, flavonoid, lactic acid bacteria, kimchi

## Abstract

Lactic acid bacteria (LAB) are generally recognized as safe (GRAS) microorganisms. This study aimed to identify novel LAB strains that can transform flavonoids into aglycones to improve bioavailability. We isolated 34 LAB strains from kimchi. The biotransformation activity of these 34 LAB strains was investigated based on α-L-rhamnosidase and β-D-glucosidase activities. Among them, 10 LAB strains with high activities were identified by 16S rRNA sequencing analysis. All tested LAB strains converted hesperidin to hesperetin (12.5–30.3%). Of these, only the *Lactobacillus pentosus* NGI01 strain produced quercetin from rutin (3.9%). The optimal biotransformation conditions for the *L. pentosus* NGI01 producing hesperetin and quercetin were investigated. The highest final product concentrations of hesperetin and quercetin were 207 and 78 μM, respectively. Thus, the *L. pentosus* NGI01 strain can be a biocatalyst for producing flavonoid aglycones in the chemical and food industries.

## 1. Introduction

Biotransformation is a chemical modification of substances by organisms and enzymes. Whole-cell systems and isolated enzymes are widely used as biocatalysts in the chemical and food industries. Biotransformation is thought to be an eco-friendly and cost-effective alternative to conventional chemical synthesis [1,2].

Flavonoids are polyphenolic secondary metabolites with diverse chemical structures found in plants. They have been reported to have beneficial effects on human health, including antioxidant, anti-inflammatory, and anti-cancer activities [3,4,5]. Ingested flavonoids are metabolized and absorbed in the small intestine, colon, and liver; they are absorbed after deglycosylation in the small intestine. Flavonoids not absorbed in the small intestine are catabolized to aglycone and phenolic acids by the microbiota and are then absorbed in the colon [6,7,8]. Generally, the aglycone forms of flavonoids are more efficiently absorbed than flavonoid glycosides because of their ability to cross through the cell membrane [9,10,11]. However, the bioavailability of most natural flavonoids is low because they are conjugated with rutinose (6-*O*-α-L-rhamnosyl-d-glucose) or neohesperidoside (2-*O*-α-L-rhamnosyl-d-glucose), and human cells lack α-rhamnosidase activity [12,13].

**Figure 1:**
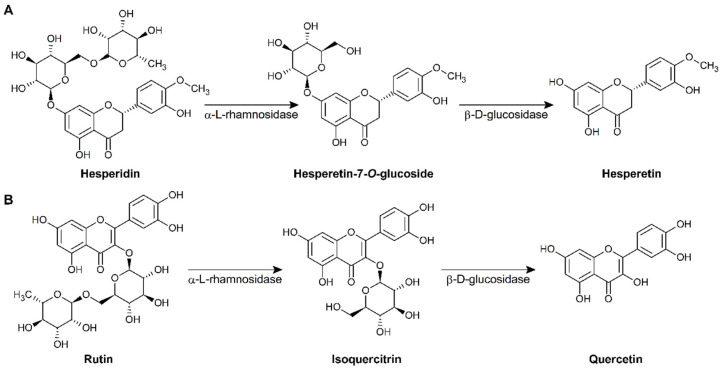
Biotransformation pathway of hesperidin (**A**) and rutin (**B**) by α-l-rhamnosidase and β-d-glucosidase.

α-l-rhamnosidase (EC 3.2.1.40) and β-d-glucosidase (EC 3.2.1.21) are glycosyl hydrolases that cleave terminal α-L-rhamnose and β-d-glucose, respectively, from several natural flavonoids [14,15,16]. For example, α-l-rhamnosidase converts hesperidin and rutin to hesperetin-7-*O*-glucoside and isoquercitrin, respectively. β-d-glucosidase then hydrolyzes these glycosides to hesperetin and quercetin, respectively (Figure 1). In the food industry, these enzymes have been applied for de-bittering fruit juices and enhancing the aroma of wine [17,18]. In addition, they are used in the production of flavonoid aglycones to increase bioavailability [10,19].

Lactic acid bacteria (LAB) are Gram-positive bacteria that produce lactic acid as the major end-product of carbohydrate fermentation. They also catabolize proteins and lipids during fermentation. LAB play important roles in the fermentation processes of foods such as cheese, yogurt, kimchi, and chongkukjang. Kimchi is a Korean tradition food, and LAB are used for kimchi fermentation [20,21,22]. LAB are generally recognized as safe (GRAS) microorganisms and can be used as safe biocatalysts. LAB strains have α-l-rhamnosidase and β-d-glucosidase activities involved in the deglycosylation of secondary metabolites during fermentation. Biotransformation using LAB has been applied for the hydrolysis of flavonoid glycosides based on these enzyme activities [13,23,24,25].

In the current study, LAB strains were isolated from kimchi, a traditional Korean fermented food, to develop novel LAB strains that could improve the bioavailability of flavonoids and be used as biocatalysts. The potential of LAB strains to transform flavonoids was evaluated by screening for the glycosyl hydrolase activity. In addition, LAB strains with a high glycosyl hydrolase activity were used as whole-cell biocatalysts for the biotransformation of hesperidin and rutin.

## 2. Materials and Methods

### 2.1. Materials

Buffered peptone water and De Man, Rogosa, and Sharpe (MRS) medium were purchased from Difco (Sparks, MD, USA). MRS medium without glucose was obtained from KisanBio (Seoul, Korea). Bromocresol purple, *p*-nitrophenyl β-d-glucopyranoside (pNPG), *p*-nitrophenyl α-l-rhamnopyranoside (pNPR), dimethyl sulfoxide (DMSO), hesperidin, hesperetin, rutin, and quercetin were purchased from Sigma-Aldrich (St. Louis, MO, USA). All chemicals were of analytical grade.

### 2.2. Isolation of LAB Strains from Kimchi

The cabbage kimchi and cucumber kimchi samples (homemade; 1 g) were cut with sterilized scissors and homogenized in 10 mL of buffered peptone water using BagMixer (Interscience, Saint-Nom la Bretèche Arpents, France). The homogenized sample was serially diluted to 10^−5^ in peptone water, and 100 μL of the dilution was spread on an MRS agar plate containing 0.0015% (*w*/*v*) bromocresol purple. After incubating at 30 °C for 24 h, yellow-colored colonies were selected. The colonies were inoculated into 10 mL of MRS medium and cultured at 30 °C until reaching an absorbance of 1 at 600 nm, which corresponded to 5 × 10^8^ colony forming unit per mL (CFU/mL). The LAB strain stocks were prepared from these cultures and stored at −80 °C.

### 2.3. Glycosyl Hydrolase Activity Assay

The α-L-rhamnosidase and β-d-glucosidase activities of LAB strains isolated from kimchi were measured by following the protocol described in a previous study [26], with modifications. The cell stock (100 μL) was inoculated into 10 mL of MRS medium without glucose, supplemented with 1 mM pNPR or pNPG. After incubating at 30 °C for 3 days, the cultures were centrifuged at 3500× *g* for 20 min, and supernatants were used for the enzyme activity assay. The supernatant (2 mL) was mixed with 5 mL of 67 mM potassium phosphate buffer (pH 6.8) and 8 mL of 100 mM sodium carbonate. The absorbance of these mixtures was measured at 405 nm using an Epoch micro plate spectrophotometer (Biotek Instruments Inc., Winooski, VT, USA). The molar extinction coefficient for *p*-nitrophenol (pNP) at 405 nm was calculated to be ε = 18.3 mM^−1^ cm^−1^ [27]. The whole cells’ glycosyl hydrolase activities were presented as the final concentrations of the product (pNP, μM).

### 2.4. Identification of LAB Strains Isolated from Kimchi

The LAB strains isolated from kimchi were identified by 16S rRNA sequencing analysis. The cell stock was inoculated into MRS broth and incubated at 30 °C for 2 days. The cultures were sent to Macrogen (Seoul, Korea) for sequencing analysis. Genomic DNA of the LAB strains was amplified using two PCR primers (27F: 5′-AGAGTTTGATCMTGGCTCAG-3′; 1492R: 5′-TACGGYTACCTTGTTACGACTT-3′). The PCR product was purified using a Montage PCR Clean up kit (Millipore, Burlington, MA, USA). The purified PCR product was sequenced using two sequencing primers (85F: 5′-GGATTAGATACCCTGGTA-3′; 907R: 5′-CCGTCAATTCMTTTRAGTTT-3′). Sequencing was performed on the Big Dye Terminator Cycle Sequencing Kit v.3.1 (Applied BioSystems, Foster City, CA, USA). Sequencing products were resolved on an Applied Biosystems model 3730XL automated DNA sequencing system (Applied BioSystems, Foster City, CA, USA) at Macrogen. The 16S rRNA sequences of LAB strains were compared with data in GenBank using the Basic Local Alignment Search Tool (BLAST).

### 2.5. Biotransformation of Hesperidin and Rutin by LAB Strains

To investigate whether the LAB strains could convert hesperidin and rutin to their aglycone forms, the cell stocks (100 μL) were inoculated into MRS medium without glucose, supplemented with 1 mM hesperidin or rutin. After incubating at 30 °C for 7 days, the cultures were freeze-dried using a freeze dryer (Operon, Gimpo, Korea). The dried samples were dissolved in 10 mL of DMSO and then filtered using a 0.2 μm GHP syringe filter (Pall Corporation, Port Washington, NY, USA). The samples were analyzed using a 1260 DAD VL detector on an Agilent 1260 Infinity HPLC system (Agilent Technologies, Santa Clara, CA, USA). The samples of 20 µL were injected into an SB-C18 column (150 × 4.6 mm, 5 μm; Agilent Technologies, Santa Clara, CA, USA). The mobile phases were 0.1% formic acid in water (A) and 0.1% formic acid in acetonitrile (B). The samples were separated via a binary program with 5% B (0–1 min), 25% B (1–30 min), 30% B (30–34 min), 30% B (34–36 min), 53% B (36–50 min), 100% B (50–51 min), and 100% B (51–55 min) at a flow rate of 1 mL/min. Hesperidin and hesperetin were detected at 280 nm. Rutin and quercetin were detected at 340 nm. The retention times of hesperidin, hesperetin, rutin, and quercetin were 26.7, 42.6, 21.9, and 34.8 min, respectively (Figure S1). The molar amounts of substrates and products were calculated from the peak area of the standards. The product yield (%) was calculated using Equation (1) [28]:

(1) $$\mathrm{Product}\;\mathrm{yield}\;\left(\%\right)=\frac{\;\mathrm{molar}\;\mathrm{amount}\;\mathrm{of}\;\mathrm{product}\;(\mathrm{mol})}{\mathrm{initial}\;\mathrm{molar}\;\mathrm{amount}\;\mathrm{of}\;\mathrm{substrate}\;(\mathrm{mol})}\;\times \;100$$

### 2.6. Production of Hesperetin and Quercetin by L. pentosus NGI01 Strain

Biotransformation of hesperidin and rutin by NGI01 was performed at different concentrations. The cell stock (100 uL) was inoculated into MRS medium without glucose, supplemented with 100–400 μM hesperidin or rutin. After biotransformation at 30 °C for 0–21 days, the cultures were freeze-dried. The products were analyzed using HPLC as described above.

### 2.7. Statistical Analysis

All experiments were performed three times. The values are presented as the mean and standard error of the mean (SEM) from values of the replicates for each sample. Statistical analysis of the experimental results was carried out using Prism 5 (GraphPad Software, San Diego, CA, USA).

## 3. Results

### 3.1. Glycosyl Hydrolase Activity of LAB Strains from Kimchi

**Figure 2:**
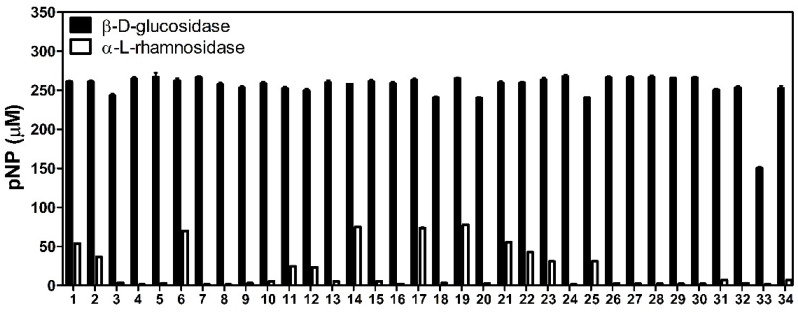
α-l-rhamnosidase and β-d-glucosidase activities of 34 LAB strains isolated from kimchi.

To identify strains that can biotransform flavonoids, we isolated 34 LAB strains from cabbage kimchi and cucumber kimchi. The α-l-rhamnosidase and β-d-glucosidase activities of isolated LAB strains were investigated using pNPR and pNPG, respectively, as substrates. The LAB strains were inoculated in MRS medium without glucose, supplemented with 1 mM pNPR or pNPG. After 3 days, the pNP produced by strains in the culture was measured. All isolated LAB strains produced over 150 μM pNP from pNPG. Among them, the NGI24 strain exhibited the highest activity, 268 μM. When pNPR was the substrate, 12 LAB strains (NGI01, NGI02, NGI06, NGI11, NGI12, NGI14, NGI17, NGI19, NGI21, NGI22, NGI23, and NGI25) produced pNP, and the NGI19 strain exhibited the highest activity, 78 μM (Figure 2).

### 3.2. Identification of LAB Strains from Kimchi

Among 34 LAB strains isolated from kimchi, 10 strains (NGI01, NGI02, NGI06, NGI14, NGI17, NGI19, NGI21, NGI22, NGI23, and NGI25) produced over 30 μM pNP from pNPR by α-l-rhamnosidase activity and more than 150 μM pNP from pNPG by β-d-glucosidase activity. Thus, these were selected for identification. Based on 16S rRNA sequencing analysis, NGI01, NGI06, NGI17, NGI19, NGI22, and NGI23 belonged to *L. pentosus*. NGI02, NGI14, NGI21, and NGI25 belonged to *L. senmaizukei*, *L. plantarum*, *L. paraplantarum*, and *L. curvatus*, respectively. The GenBank database accession numbers of the LAB strains are shown in Table 1.

### 3.3. Biotransformation of Hesperidin and Rutin by the L. pentosus NGI01 Strain

To investigate whether the 10 LAB strains could convert hesperidin and rutin to their aglycone forms, strains were cultured in MRS medium without glucose, supplemented with 1 mM hesperidin or rutin for 7 days. After whole-cell biotransformation with LAB, the yield of hesperetin and quercetin was analyzed by HPLC. All tested LAB strains converted hesperidin to hesperetin. Of these, the NGI01 strain produced the most hesperetin, with a yield of 30.3%. The hesperetin yield of the NGI06 strain was the second highest, 28.3%. The NGI23 strain yielded the least hesperetin, with a yield of 12.5%. When rutin was present in the culture, only NGI01 produced quercetin from rutin, with a product yield of 3.9% (Table 2).

### 3.4. Production of Hesperetin and Quercetin by L. pentosus NGI01 Strain

**Figure 3:**
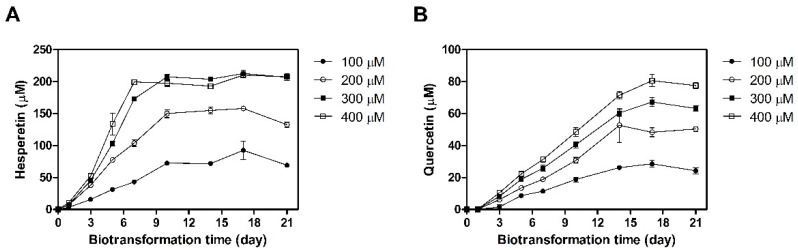
Production of hesperetin (**A**) and quercetin (**B**) by *L. pentosus* NGI01.

The optimal conditions to produce hesperetin and quercetin using the NGI01 strain were determined by varying the substrate concentration and biotransformation time. Hesperetin production increased with increasing hesperidin concentration and biotransformation time, reaching saturation after 10 days. The NGI01 strain produced the highest concentration of hesperetin, 207 μM, from 300 μM hesperidin after 10 days. The product yield of hesperetin under these conditions was 69.1% (Figure 3A). In addition, the NGI01 strain converted rutin to quercetin. Quercetin production increased with rutin concentration and biotransformation time and was saturated within 17 days. The most quercetin produced was 78 μM, obtained when rutin concentration was 400 μM, and the production yield of quercetin was 19.4% (Figure 3B). Thus, the optimal biotransformation conditions for producing hesperetin by the NGI01 strain were: MRS medium without glucose, supplemented with 300 μM hesperidin, at 30 °C, and for 10 days. The optimal conditions for quercetin production by NGI01 were: MRS medium without glucose, supplemented with 400 μM rutin, at 30 °C, and for 17 days.

## 4. Discussion

Flavonoids have a potential as biomaterials in the development of functional foods and medicines because they have beneficial effects on human health [4,5]. Most natural flavonoids are glycoside forms such as rutinose and neohesperidoside. To improve the bioavailability of flavonoids, ingestion of flavonoids in aglycone forms or activities of gut microbiota are necessary because α-rhamnosidase activity is absent in humans [8,13]. Since LAB are GRAS microorganisms and have both α-l-rhamnosidase and β-d-glucosidase activities [25], they can be biocatalysts for biotransformation of flavonoids to aglycones.

Here, LAB strains with high glycosidase activity were isolated from kimchi, a Korean traditional food, and identified by 16S rRNA sequence analysis (Table 1). All LAB strains exhibited β-d-glucosidase activity and 12 LAB strains showed α-l-rhamnosidase activity. The β-d-glucosidase activity was higher than α-l-rhamnosidase activity in tested LAB strains (Figure 2). These results suggest the LAB strains isolated from kimchi have a potential as whole-cell biocatalysts for glycosyl hydrolysis of flavonoid glucosides.

To investigate whether the LAB strains could biotransform flavonoids, hesperidin and rutin were used as substrates in whole-cell biotransformation. After whole-cell reactions, 10 LAB strains produced hesperetin from hesperidin and only the NGI01 strain produced quercetin from rutin (Table 2). We also tested whether naringin was converted to naringenin by the 10 LAB strains, but no naringenin was produced by the strains (data not shown). These results suggested that LAB strains isolated from kimchi showed different substrate specificity [16,29,30]. In addition, hesperetin-7-*O*-glucoside and isoquercitrin, products of α-L-rhamnosidase (Figure 1), were not detected in the whole-cell reactions using NGI01 (Figure S1). These results are related to Figure 2 which shows that the β-d-glucosidase activity of LAB strains was higher than α-l-rhamnosidase activity.

A previous study described the biotransformation of hesperidin and rutin using probiotics such as *L. plantarum* and *L. acidophilus* LA-5. When the concentration of hesperidin and rutin was 100 μM, the conversion rates of the two by *L. acidophilus* were 84% and 3%, respectively, after 10 days. *L. plantarum* yielded 70% hesperetin and 5% quercetin after 10 days [13]. In this study, the product yields of hesperetin and quercetin by the *L. pentosus* NGI01 strain after 10 days of biotransformation were 71.8% and 18.8% from 100 μM hesperidin and rutin, respectively (Figure 3). To investigate the stability of hesperidin and rutin, the flavonoids were incubated without whole cells for 21 days. The concentration of hesperidin was not apparently changed. Although the rutin concentration decreased over the time after 7 days of incubation, the product of biotransformation, quercetin, was not detected (Figures S2 and S3). In addition, cell growth was monitored during the NGI01-induced biotransformation. The growth of the NGI01 strain was stationary for 21 days in media containing hesperidin. In case of media containing rutin, the growth of the NGI01 strain decreased after 7 days, but quercetin, a product of biotransformation, was continuously produced after 7 days. (Figure S4 and Figure 3). These current results suggest that the *L. pentosus* NGI01 strain can produce hesperetin and quercetin with a high product yield. The *L. pentosus* NGI01 strain identified in this study was deposited at the Korean Culture Center of Microorganisms as KCCM12592P.

In conclusion, the *L. pentosus* NGI01 strain isolated from kimchi was a LAB strain and was a GRAS microorganism. This NGI01 strain had α-l-rhamnosidase and β-d-glucosidase activities. In addition, it produced high yields of hesperetin and quercetin from hesperidin and rutin, respectively. Therefore, the NGI01 strain can be applied as a safe whole-cell biocatalyst for chemical synthesis with applications in the food industry.

## Figures and Tables

**Table 1:** Identification of LAB strains with high enzyme activities.

Strain	Isolation Source	Species	Accession Number
NGI01	Cucumber kimchi	*L. pentosus*	MT898558
NGI02	Cucumber kimchi	*L. senmaizukei*	MT898562
NGI06	Cabbage kimchi	*L. pentosus*	MT898563
NGI14	Cabbage kimchi	*L. plantarum*	MT898568
NGI17	Cabbage kimchi	*L. pentosus*	MT919355
NGI19	Cabbage kimchi	*L. pentosus*	MT898576
NGI21	Cabbage kimchi	*L. paraplantarum*	MT898577
NGI22	Cabbage kimchi	*L. pentosus*	MT898642
NGI23	Cabbage kimchi	*L. pentosus*	MT898646
NGI25	Cabbage kimchi	*L. curvatus*	MT898647

**Table 2:** Biotransformation of hesperidin and rutin by 10 LAB strains isolated form kimchi.

Strain	Product Yield (%)	
	Hesperidin → Hesperetin	Rutin → Quercetin
NGI01	30.3 ± 0.1	3.9 ± 0.2
NGI02	26.3 ± 2.3	0
NGI06	28.3 ± 0.8	0
NGI14	20.4 ± 0.1	0
NGI17	26.7 ± 0.1	0
NGI19	24.9 ± 0.2	0
NGI21	22.7 ± 0.1	0
NGI22	23.1 ± 0.1	0
NGI23	12.5 ± 0.1	0
NGI25	15.5 ± 0.1	0

## Data Availability

The accession numbers in Table 1 are available in GenBank database (https://www.ncbi.nlm.nih.gov/genbank/ (accessed on 5 April 2021)). The *L. pentosus* NGI01 strain is available in Korean Culture Center of Microorganisms (http://www.kccm.or.kr/ (accessed on 5 April 2021)).

## Supplementary Materials

The following are available online at https://www.mdpi.com/article/10.3390/microorganisms9051075/s1, Figure S1: Chromatogram of flavonoids after the biotransformation by the *L. pentosus* NGI01 strain, Figure S2: Stability of hesperidin and rutin, Figure S3: Chromatograms of flavonoids after incubation without whole-cell biocatalysts, Figure S4: Growth of the *L. pentosus* NGI01 strain during biotransformation.
