# Supplementary material for: Biotransformation of Flavonoids by Newly Isolated and Characterized Lactobacillus pentosus NGI01 Strain from Kimchi
Source: Microorganisms. 2021 May 17;9(5):1075. doi: 10.3390/microorganisms9051075 (PMC8157076; doi:10.3390/microorganisms9051075)
Supplement: Supplementary file 1 [file microorganisms-09-01075-s001.zip › microorganisms-1217189-supplementary.pdf]

Supplementary Materials

Biotransformation of Flavonoids by Newly Isolated and Characterized *Lactobacillus pentosus* NGI01 Strain from Kimchi

Chan Mi Park [1], Gyoung Min Kim [2], and Gun Su Cha [2,*]

1 School of Biological Sciences and Technology, Chonnam National University, 77 Yongbongro, Gwangju 61186, Korea; cmpark0710@gmail.com
2 Department of Research and Development, Namhae Garlic Research Institute, 2465-8 Namhaedaero, Namhae 52430, Korea; policecop@hanmail.net
* Correspondence: gscha450@gmail.com; Tel.: +82-55-860-8953; Fax: +82-55-860-8960

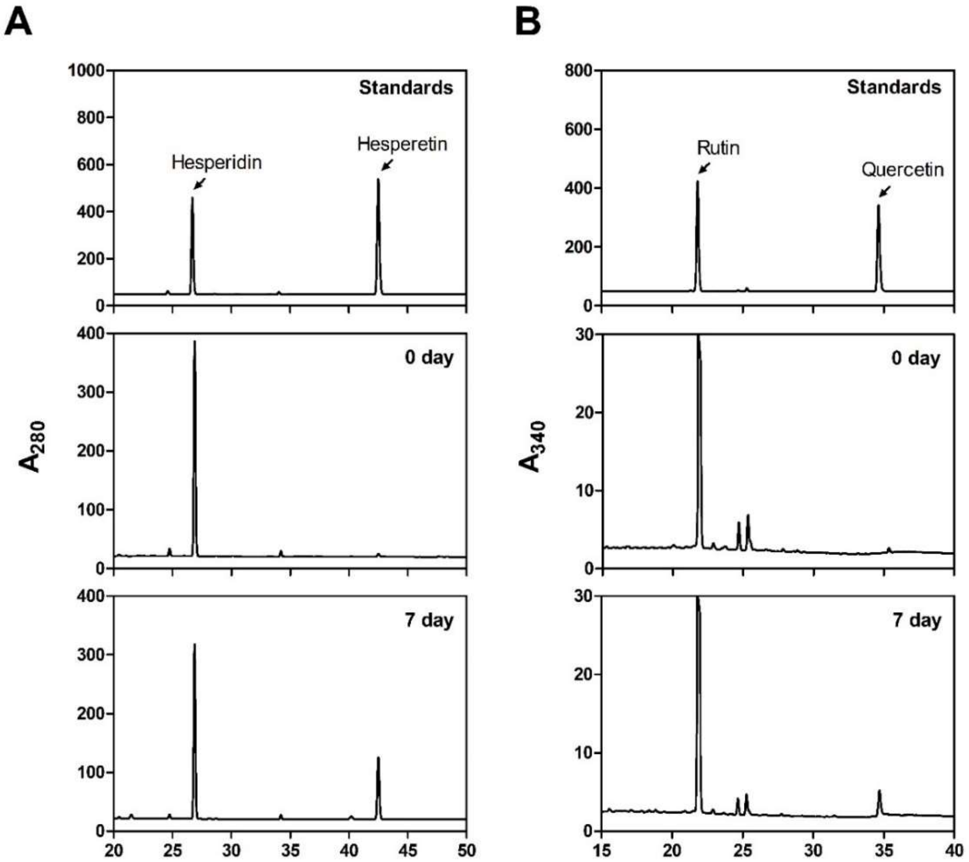

Figure S1. Chromatogram of flavonoids after the biotransformation by the *L. pentosus* NGI01 strain.

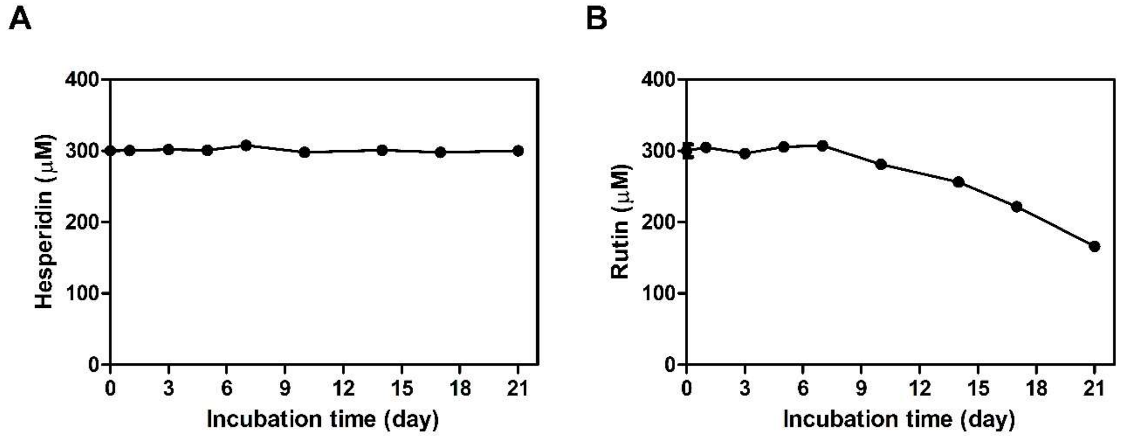

Figure S2. Stability of hesperidin and rutin. The MRS medium without glucose, supplemented with 300 μM hesperidin (A) or rutin (B). After incubation without whole cells at 30 °C for 21 days, the media were freeze-dried. The flavonoids were analyzed using HPLC.

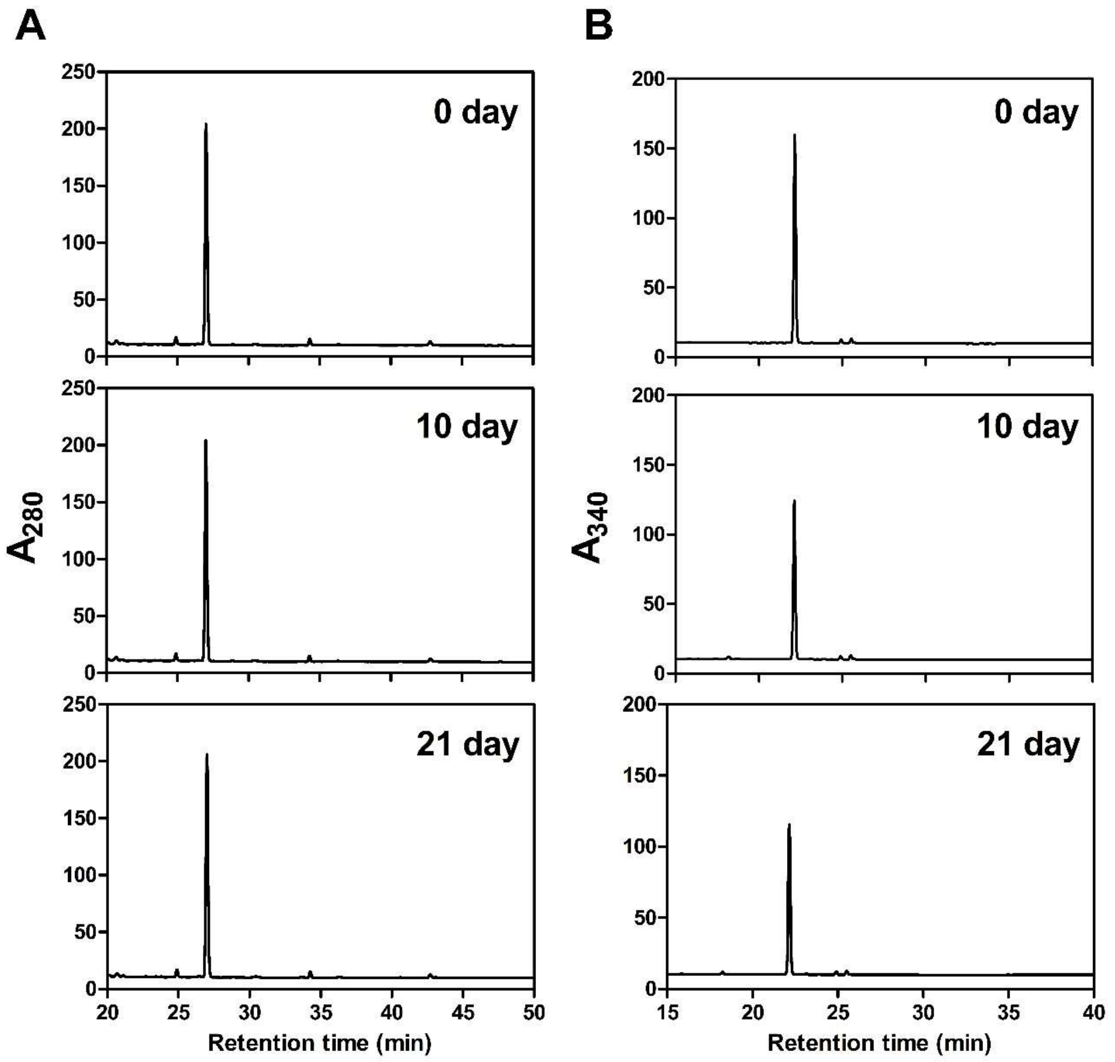

Figure S3. Chromatograms of flavonoids after incubation without whole-cell biocatalysts. The MRS medium without glucose, supplemented with 300 μM hesperidin (A) or rutin (B). After incubation without whole cells at 30 °C for 0, 10, and 21 days, the media were freeze-dried. The flavonoids were analyzed using HPLC. Hesperidin and rutin were detected at 280 nm and 340 nm, respectively. The retention times of hesperidin, hesperetin, rutin, and quercetin were 26.7, 42.6, 21.9, and 34.8 min, respectively

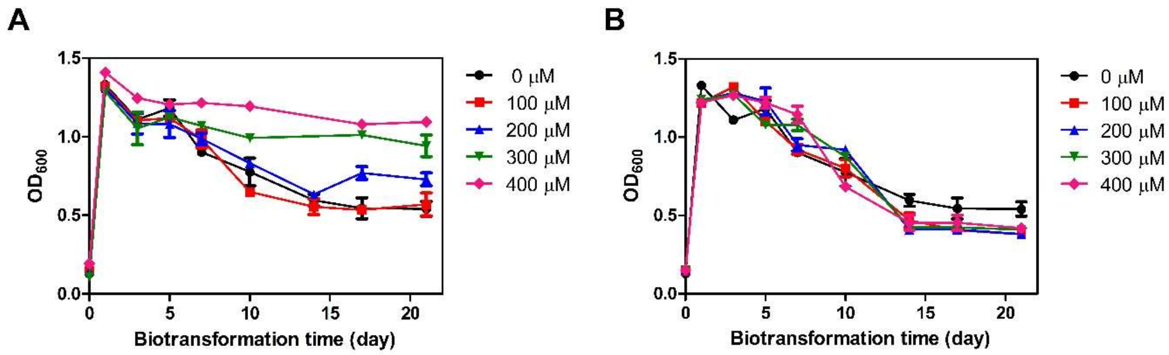

Figure S4. Growth of the *L. pentosus* NGI01 strain during biotransformation. The cell stock (100 μL) was inoculated into MRS medium without glucose, supplemented with 0–400 μM hesperidin (A) or rutin (B). After culture at 30 °C for 0–21 days, absorbance of the cultures was measured at 600 nm.
